# A Visual Fault Detection Method for Induction Motors Based on a Zero-Sequence Current and an Improved Symmetrized Dot Pattern

**DOI:** 10.3390/e24050614

**Published:** 2022-04-28

**Authors:** Liangyuan Huang, Jihong Wen, Yi Yang, Ling Chen, Guoji Shen

**Affiliations:** 1 College of Intelligence Science and Technology, National University of Defense Technology, Changsha 410073, China; huangliangyuan@nudt.edu.cn (L.H.); wenjihong@vip.sina.com (J.W.); turbineyy@163.com (Y.Y.); llcl1105@163.com (L.C.); 2 Laboratory of Science and Technology on Integrated Logistics Support, National University of Defense Technology, Changsha 410073, China

**Keywords:** induction motors, fault detection, local symmetrized dot pattern, zero-sequence current, kernel density estimation

## Abstract

Motor faults, especially mechanical faults, reflect eminently faint characteristic amplitudes in the stator current. In order to solve the issue of the motor current lacking effective and direct signal representation, this paper introduces a visual fault detection method for an induction motor based on zero-sequence current and an improved symmetric dot matrix pattern. Empirical mode decomposition (EMD) is used to eliminate the power frequency in the zero-sequence current derived from the original signal. A local symmetrized dot pattern (LSDP) method is proposed to solve the adaptive problem of classical symmetric lattice patterns with outliers. The LSDP approach maps the zero-sequence current to the ultimate coordinate and obtains a more intuitive two-dimensional image representation than the time–frequency image. Kernel density estimation (KDE) is used to complete the information about the density distribution of the image further to enhance the visual difference between the normal and fault samples. This method mines fault features in the current signals, which avoids the need to deploy additional sensors to collect vibration signals. The test results show that the fault detection accuracy of the LSDP can reach 96.85%, indicating that two-dimensional image representation can be effectively applied to current-based motor fault detection.

## 1. Introduction

Since the motor is the most widely used and efficient power source in the current industrial system, the running state of a motor is directly related to the continuity and reliability of a task chain. In large engineering fields such as mining and aerospace, it is often expensive to disassemble and inspect critical motors, and the unexpected failures of these machines can lead to even more significant losses. Therefore, motor fault detection and diagnosis have outstanding economic value and practical significance.

From the perspective of measuring signal types, some researchers have studied motor faults by analyzing physical quantities such as sound [1], temperature [2], and magnetic flux [3], but the most commonly used are vibration and current signals. Based on the principle that the mechanical fault characteristics of a motor are manifested in the vibration signal, the most popular vibration analysis method combines signal processing and feature extraction to analyze the possible fault information, which is interoperable with other types of mechanical equipment fault diagnosis methods. Classical techniques in vibration analysis include Fourier transform, wavelet transform, empirical mode decomposition (EMD), and envelope demodulation. However, since vibration data often contain complex ambient noise and other disturbances, these techniques are often suboptimal for weak fault detection. In addition, since vibration analysis can be considered to extract aperiodic signals from cyclic stationary vibration signals, it is possible to use machine learning to learn from known samples and identify faults. Therefore, vibration analysis methods are often used in conjunction with artificial intelligence techniques. In the research of the time–frequency analysis method, Yu [4] proposed a transient extraction transform based on a short-time Fourier transform that could effectively characterize and extract transient components in fault signals. Aiming at the cumbersome parameter definition of the stochastic resonance method, Cui et al. [5] proposed a coupled multi-stable stochastic resonance method with two first-order multi-stable stochastic resonance systems. They adaptively optimized and determined the system parameters of the stochastic resonance method by taking the signal-to-noise ratio as the fitness function of the search optimization algorithm using the subsampling technique. To improve the readability of the time–frequency analysis method, Zhang et al. [6] proposed a framework for improving the time–frequency post-processing method to reduce the interference of cross-terms and enhance the time–frequency energy concentration characteristic. In the field of artificial intelligence technology, Sikder et al. [7] extracted the power features in motor vibration data and introduced the extreme learning machine method to a motor-bearing fault diagnosis. The average classification accuracy rate could reach 98.67%. Furthermore, Shao et al. [8] developed an auxiliary classifier generative adversarial network-based framework for learning local features from raw inputs and generating synthetic signals with labels. These generated signals could be used as augmented data for further applications in machine learning for fault diagnosis. Nishat et al. [9] combined empirical mode decomposition and kurtosis to filter out irrelevant eigenmode functions and transformed a reconstructed signal into a two-dimensional image with a continuous wavelet transform to train the convolutional neural network model.

Motor current signature analysis (MCSA) is generally used to diagnose electrical faults in motors, such as broken rotor bars, winding short circuits, and single-phase grounding [10]. Existing research shows that MCSA can also be used to detect mechanical failures [11,12]. In contrast to the intrusive installation of vibration sensors or expensive non-contact measurements, the current signal is obtained through a sensor (such as the current clamp) connected to the power supply cable of a motor, which gives MCSA the significant advantage of low signal acquisition cost and integration with the control system. MCSA mainly inspects the amplitude and phase changes of the stator current caused by motor failure components, such as waveform distortion, so its data processing and analysis methods are analogous to vibration analysis. Corne et al. [13] studied the mapping relationship between three types of bearing evolution faults and a motor current and analyzed this relationship by extending the Park vector method. Based on the magnetic field theory, Khelfi et al. [14] demonstrated that a broken rotor bar induced an amplitude modulation of the stator current that could be extracted with a low computational effort by calculating the square root of the three-phase stator current. Abd-El-Malek et al. [15] used a Hilbert transform to extract fault features from the stator current envelope and perform a statistical analysis to generate a formula to obtain the exact location of the fault in the induction machine rotor. For large induction motors with a low slip rate, Puche-Panadero et al. [16] proposed the use of the spectrum of the rectifier stator current for fault diagnosis to eliminate the coverage of the fundamental wave leakage to the early fault components. Li et al. [17] used the Teager–Kaiser energy operator to obtain the estimation of the instantaneous amplitude and instantaneous frequency of a motor current signal, to remove the main power component of a motor current and highlight the broken rotor bar fault feature. The classic vibration envelope analysis is also used for reference in MCSA. Areias et al. [18] proposed the identification of the fault characteristic frequency of rolling bearings in the current spectrum by performing an envelope analysis on the bearing vibration signal. MCSA can also be combined with artificial intelligence methods. Garcia-Bracamonte et al. [19] applied independent component analysis to the Fourier domain spectral signals of a single-phase current. The segmented standard deviation in the region of interest was used as the input feature of a neural network to diagnose an induction motor broken bar fault.

Based on the existing research, no matter what type of signal is selected, it is difficult to achieve an intuitive visual representation in the signal processing and feature extraction stages, which is difficult for untrained diagnostic system users to understand. Therefore, the emergence of image analysis technology provides a new method for the simple visual representation of fault features, and it provides the possibility to improve the usability of fault detection and diagnosis systems. The traditional form of converting time-domain signals into two-dimensional images involves time–frequency images, and this form of expression still has a certain threshold for objective understanding. The Symmetrized Dot Pattern (SDP), which is based on a fixed time delay, can linearly map a one-dimensional time series signal to a two-dimensional symmetric snowflake scatter image with a relatively small amount of computation and express signal changes with obvious visual changes. Several studies have shown the effectiveness of the SDP for the visual analysis of motor vibration signals. For example, Wang et al. [20] used the SDP method to map the bearing vibration data into a picture data set, and they input SE-CNN training to obtain a diagnostic model. Sun et al. [21] performed empirical modal decomposition on a motor-bearing vibration data set and then used the first five intrinsic mode functions components to draw an SDP image and calculate an improved distance metric for fault classification. Combining SDP images and dense Scale Invariant Feature Transform (SIFT) features, Long et al. [22] proposed a method for motor fault diagnosis based on the visual image information and the bag-of-words model.

Considering the advantages of MCSA compared to vibration signals in terms of acquisition cost and system complexity, for the visual analysis of current signals, the SDP method has the potential to be popularized and widely applied. However, due to the low ratio of the characteristic amplitude of the current signal to the amplitude of the fundamental frequency, it is impractical to use the SDP image analysis of a current directly, so there is still a gap in present research on the SDP combined with the motor current. Furthermore, the SDP method is less robust with large noise glitches, which is one of the reasons why it is unfavorable to use it for analyzing current signals. For the scatter images obtained by SDP and its derivative methods, the KDE method offers the possibility to add density information to them. Given the above considerations, this paper proposes a fault detection method based on the LSDP and KDE color mapping that utilizes the fault features implicit in the zero-sequence current distribution to distinguish between normal and fault samples. That is to say, this method can overcome the effect of the current fundamental frequency to plot a more robust scatter plot than the traditional SDP method. Furthermore, compared to many fault detection methods based on Fourier transforms, this method does not require a large number of complex calculations and has a fixed procedure, making it valuable for designing integrated circuits for use in embedded devices. The method proposed in this paper has a general mathematical framework and is not restricted to specific software and programming environments. The main work is presented as follows:

1. The EMD method is used to eliminate the fundamental and low-frequency components in the zero-sequence current, preserving the fault components in the current signal and enhancing the representation of the difference between the normal and fault samples.
2. The LSDP method is proposed to draw the zero-sequence current image to improve the stability of the two-dimensional scatter image.
3. Combining the SDP and the LSDP with KDE enhances the visual information representation difference to draw scatter images with colormaps. Through the test, the effectiveness of the LSDP in fault detection is verified.

The rest of this paper is organized as follows. The second section briefly introduces the method adopted in this paper and its rationale. The third section introduces the experimental bench setup and discusses the relevant results. The fourth section states the conclusions.

## 2. Basic Method Framework

### 2.1. Zero-Sequence Current

Ideally, an induction motor is a symmetrical electromechanical system, and the three-phase currents are equal in amplitude and 120° out of phase during a load operation. When part of the motor fails, it will cause fluctuations in the physical characteristics such as the load, impedance, and magnetic flux, which implies that the parameter balance of the induction motor will be broken. The asymmetry of the three-phase current is the main manifestation of the motor fault in the phase current.

**Figure 1:**
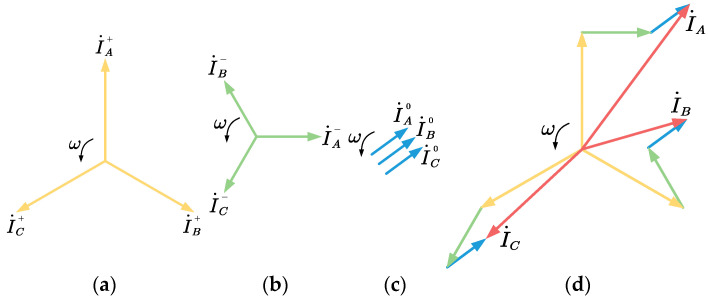
Three-phase mode decomposition. (**a**) Positive-sequence components; (**b**) negative-sequence components; (**c**) Zero-sequence components; (**d**) Synthetic three-phase mode.

Existing research shows that any three-phase mode can be decomposed into a unique set of positive-sequence components, negative-sequence components, and zero-sequence components, which indicates that any three-phase mode can be represented by this group of composite bases. As shown in Figure 1, in an ideal three-phase power system, due to the symmetry of the three phases, the amplitudes of the negative-sequence component and the zero-sequence component are both zero. That is, only the positive-sequence component exists. Theoretically, only the three-phase model of the system with faults is unbalanced, and the non-zero negative sequence and zero-sequence components can be decomposed at this time.

It is almost impossible for a motor to achieve perfect parametric symmetry in practical applications. In addition, with the noise and error of measurement, the negative sequence current and the zero-sequence current are always objective and non-zero in value. In addition to the noise caused by measurement, the zero-sequence and negative-sequence currents reflect the fault state of a motor. Zero-sequence currents are sensitive to ground faults and contain weak features that reflect other faults.

### 2.2. Empirical Mode Decomposition

EMD is an adaptive method for analyzing non-stationary signals. According to the features of different scales in the time-series signal, the method separates the original signal into signal components of different scales, called intrinsic mode functions (IMFs). IMFs have two constraints:

- In the entire data segment, the number of extreme points and zero-crossing points must be equal, or the difference cannot exceed one.
- At any time, the average value of the upper envelope formed by the local maximum points and the lower envelope formed by the local minimum points is zero. That is, the upper and lower envelopes are locally symmetrical with respect to the time axis.

The basic steps of EMD processing for time-series signals are as follows:

1. All of the extreme points of the original signal *x*(*t*) are found and the upper and lower envelopes pass through these extreme points according to the cubic spline function.
2. The mean curve *m*_1_ of the upper and lower envelopes is calculated.
3. The mean curve is subtracted from the original signal to obtain the intermediate signal *h*_1_ = *x*(*t*) − *m*_1_.
4. It is judged whether the intermediate signal *h*_1_ satisfies the two constraints of the IMF. If the constraints are satisfied, the signal is an IMF component. Otherwise, based on the signal, the analysis of steps 1 to 4 is performed again. The acquisition of IMF components usually requires several iterations.
5. The first IMF obtained using the above method is denoted as *c*_1_, the difference between the original signal *x*(*t*) and *c*_1_ is used as the new original signal *r*_1_ = *x*(*t*) − *c*_1_, and the analysis of steps 1 to 4 is continued. Then *c*_2_ can be obtained. This continues until the residual signal of the nth order is decomposed into a monotonic function, the IMF component can no longer be sieved, and the EMD decomposition is completed.

Compared with other time–frequency domain methods such as wavelet transform, the EMD method decomposes adaptively by relying on the characteristics of the signal itself, which overcomes the lack of self-adaptability of a given basis function. The IMF obtained by decomposition contains the frequency components of the original signal from high to low. Due to the inherent asymmetry of the motor and power system, the zero-sequence current also contains a large frequency component of the power frequency and lower frequency. By removing the IMFs of the irrelevant frequencies in the original signal, the interference caused by the power frequency and the low frequency can be further reduced, and the leakage of the signal energy can be effectively avoided at the same time.

### 2.3. Local Symmetrized Dot Pattern

**Figure 2:**
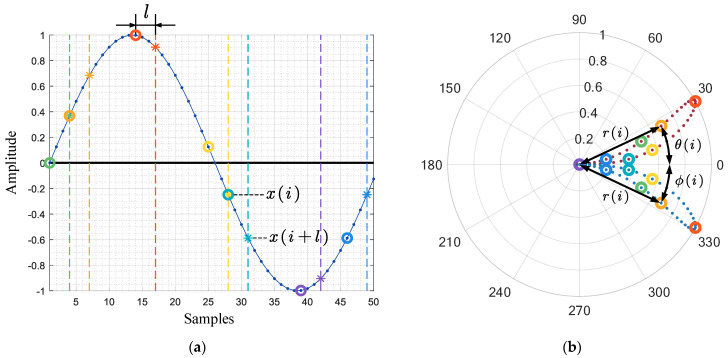
The principle of SDP. (**a**) Time−series signal before SDP transformation; (**b**) Scatter diagram after SDP transformation.

The SDP method was first proposed by Pickover [23] and applied to the visual representation of speech signals. In contrast to time–frequency analysis, this algorithm maps the normalized time waveform to a symmetric point space, creating a scatter plot related to the amplitude and frequency of the time series signal on a polar plot, as shown in Figure 2. Therefore, as a method to convert a one-dimensional time series signal into a two-dimensional graph, the SDP method can represent the changes in the amplitude and frequency and the characteristics of the distribution of the time series signals in a more understandable visual form, which provides a new perspective for fault diagnosis without frequency domain analysis. Its definition formula is as follows [23]:

(1) $$r(i)=\frac{x_{i}-x_{min}}{x_{max}-x_{min}}$$

(2) $$\Theta (i)=\theta +\frac{x_{i+l}-x_{min}}{x_{max}-x_{min}}g$$

(3) $$\Phi (i)=\theta -\frac{x_{i+l}-x_{min}}{x_{max}-x_{min}}g$$

In the expression, *r*(*i*) is the radius of the *i*th point in polar coordinates, *x_i_* is the amplitude of the *i*th sampling point of the original signal, *x_min_* is the minimum value of the original signal, *x_max_* is the maximum value of the original signal, *Θ*(*i*) is the clockwise deflection angle of the *i*th point along the mirror symmetry plane in polar coordinates, *l* is the delay coefficient, *θ* is the rotation angle of the mirror symmetry plane, and *g* is the deflection angle gain.

**Figure 3:**
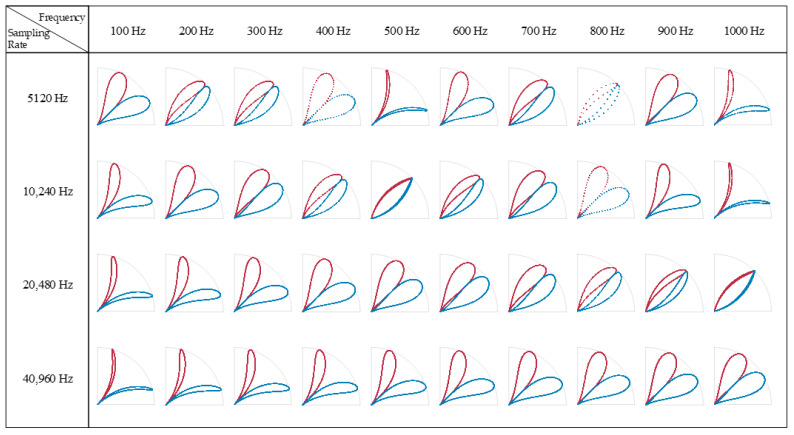
SDP representation of single−frequency signals at different frequencies.

**Figure 4:**
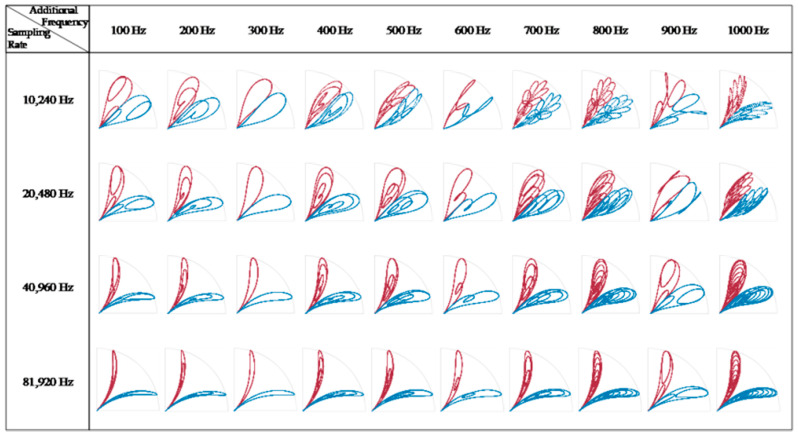
SDP representation of dual−frequency signals with different frequencies.

The breakthrough of the SDP is that a consistent time lag is adopted, with the consideration that the signal autocorrelation feature may appear as a characteristic pattern at a certain lag. However, regarding the choice of parameters, there is still a lack of established methods for determining the appropriate SDP parameters in the existing literature, so the trial-and-error method is still the most direct way to obtain stable patterns. At present, most of the research literature on SDP methods for vibration and sound signals recommends that the value of the delay coefficient should not exceed 10 [24]. However, due to the consideration of the new object to study the current signal and high sampling rate, the selection of this study is not limited to the suggested range. After several experiments, it is found that when *l* is around 1000, the image morphology is the best and is insensitive to small-scale changes, so the following research takes *l* = 1000. The SDP of a single sample signal appears as a pair of scatter arms symmetrical about the *θ* angle, which occupies only a fraction of the area in polar coordinates. To facilitate the study of the signal characteristics, either the scatter arm of a single sample is generally rotated multiple times to cover the entire coordinate area, or multiple sample signals are drawn at different *θ* angles. The differences in the SDP images are mainly reflected in the following aspects: (i) The curvature change of the scatter arm. (ii) The thickness and shape characteristics of the scatter arm. (iii) The geometric center of the scatter arm. (iv) The concentrated area of the scatter. These differences are mainly affected by factors such as parameter settings, sampling frequency, signal frequency, and noise distribution. Taking a single frequency sinusoidal signal as an example, the SDP parameters *l* = 10, $\theta =45^{\circ }$, $g=30^{\circ }$ are set. As shown in Figure 3, for a sinusoidal signal *y* = sin(2*πft*) with a fixed sampling frequency *fs*, the same image repeatedly appears as the signal frequency *f* increases. There are differences in the SDP images presented by the same frequency at different sampling frequencies. Overall, for the case of *f < fs*, the scale of the SDP image changes with the increase in the signal frequency *f* is negatively correlated with the value of *fs*. For a multi-frequency signal *y* = sin(2*π* ∗ 300 ∗ *t*) + sin(2*πft*), the distribution pattern of the SDP image is significantly affected by the frequency *f* of the superimposed signal, as shown in Figure 4. For the case of a low sampling rate, the distribution pattern of the SDP image has no obvious regularity with the increase in superposition frequency *fs*. Nevertheless, for the case of a high sampling rate, the width and the internal distribution of the scatter arms show a trend with the increase in the superposition frequency, which is beneficial for the visual analysis of the signal.

During the experiment, it was found that due to the low signal-to-noise ratio in the zero-sequence current, a few samples have some sampling points that deviate from the overall distance and cause instability in the radial range of the scattered concentrated area of the classic SDP image. Thus, a local symmetrized dot pattern method is proposed. By setting the length of the calculation point correlation window, the oscillation range of the scattered point concentration area is limited, and the influence of the direct removal of wild points on the adaptability of the SDP method is avoided. The definition formula is as follows:

(4) $$X(i)=\left\{x_{j}|i-\frac{h}{2}\leq j\leq i+\frac{h}{2},j\in {\mathbb{N}}^{+}\right\}$$

(5) $$r_{L}(i)=\frac{x_{i}-\min(X(i))}{\max(X(i))-\min(X(i))}$$

(6) $$\Theta_{L}(i)=\theta +\frac{x_{i+l}-\min(X(i))}{\max(X(i))-\min(X(i))}g$$

(7) $$\Phi_{L}(i)=\theta -\frac{x_{i+l}-\min(X(i))}{\max(X(i))-\min(X(i))}g$$

where *h* is the window length, *i* is the sampling point number with $i\in \left[1+\frac{h}{2},L-\frac{h}{2}\right]$, *L* is the sample length, and *X*(*i*) is the set of all sampling points in the *i*th sampling point window.

**Figure 5:**
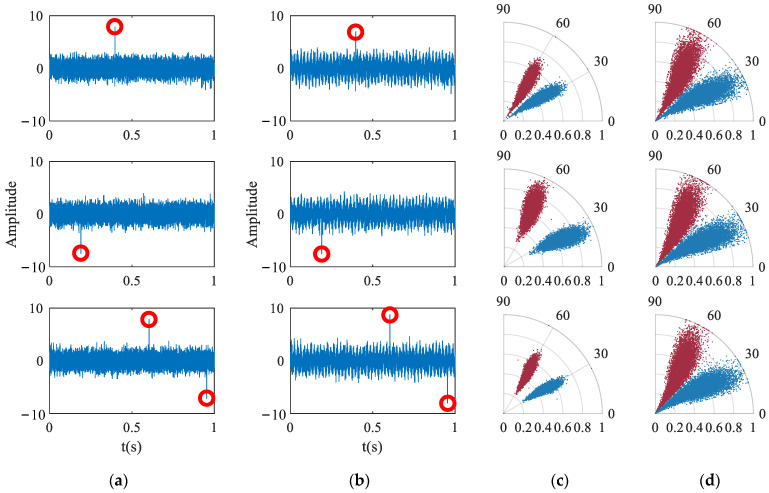
Comparison of SDP and LSDP. (**a**) Noise with outliers; (**b**) Simulated zero sequence current with outliers; (**c**) SDP image; (**d**) LSDP image.

The zero-sequence current is simulated to construct a noisy single frequency signal *y* = sin(2*π* ∗ 50 ∗ *t*), where the noise is the Gaussian noise with a mean of zero and a standard deviation of 1. Sampling points with large deviation values are inserted into the noise signal, as shown in Figure 5. It can be seen from the figure that the scatter distribution of the classic SDP image is greatly offset by the interference of wild points, and the local SDP method can better avoid this effect.

### 2.4. Scatter Density Mapping Based on Kernel Density Estimation

Due to the limitation of the drawing method, the general SDP scatter image loses the information for the distribution situation in the overlapping area of the scatter. In this study, the density is mapped to a color map utilizing the scatter probability density estimation to obtain a richer representation of the SDP visual information. KDE is a nonparametric test method for estimating unknown density functions [25,26]. In contrast to parametric methods, KDE methods are not affected by parametric model specification problems, and these methods can obtain accurate estimates of existing samples without any distribution assumptions. The algorithm calculates the adaptive kernel function bandwidth according to the given sample point set, considers the contribution of other points in the neighborhood to the probability density of each point *x* in the bandwidth, and then solves the probability density function of the sample distribution.

For a set of independent and identically distributed sample points $X=\left\{x_{1},x_{2},x_{3},\ldots ,x_{i}\right\}$, the density estimate *x* is given by [27]:

(8) $$\hat{f}(x)=\frac{1}{nh}\sum_{i=1}^{n}K\left(\frac{x-x_{i}}{h}\right)$$

where *h*(*h* > 0) is the bandwidth length, *K*(*x*) is the kernel function, and *x_i_* is the *i* sample point. *K*(*x*) has to satisfy the following conditions:

(9) $$\{\begin{array}{ccc} K\left(x\right) & \geq & 0\\ \int K\left(x\right)dx & = & 1\\ \int xK\left(x\right)dx & = & 0\\ \int x^{2}K\left(x\right)dx & > & 0 \end{array}$$

Although the KDE method has the problem of kernel function selection, theoretically, the probability density estimation obtained by any kernel function form is reliable when the number of samples is large enough. Relevant research specifies that different kernel functions have little effect on the asymptotic characteristics of the estimator, so the choice of kernel function is not the focus of KDE. Since the current measurement noise approximates a Gaussian distribution, the Gaussian kernel function is selected as the kernel function in this work. The following equations give the kernel function and the KDE function.

(10) $$K(x)=\frac{1}{\sqrt{2\pi }}\exp(-\frac{1}{2}x^{2})$$

(11) $$\hat{f}(x)=\frac{1}{nh}\sum_{i=1}^{n}\frac{1}{\sqrt{2\pi }}\exp\left(-\frac{{\left(x-x_{i}\right)}^{2}}{2h^{2}}\right)$$

For the SDP image, the probability density estimate corresponding to each point is obtained by combining the *r*(*i*) obtained from Equations (1)–(3) with *Θ*(*i*) and *Φ*(*i*) into a bivariate matrix and inputting this into Equation (11). The colormap maps each scatter point to a polar plot with a distinguishable color to obtain a visual representation of the density of the SDP scatter point image.

### 2.5. Proposed Method

**Figure 6:**
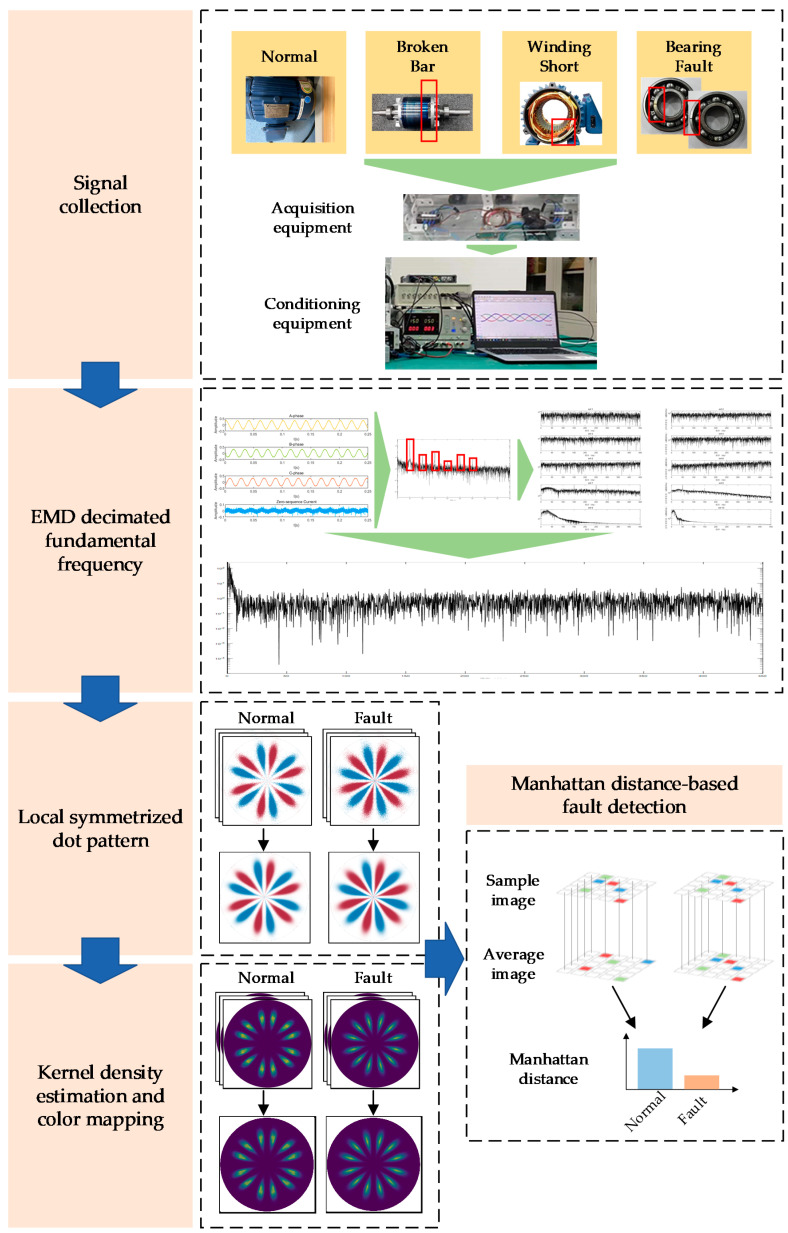
Flow chart of visual fault detection method based on zero-sequence current and LSDP.

By combining the aforementioned basic principles, this paper proposes a visualization method for fault detection based on LSDP and zero-sequence current power frequency elimination through EMD. The flowchart is shown in Figure 6, and it mainly includes the following steps:

1. The original signals of the three-phase current of the induction motor under different working conditions are acquired.
2. The zero-sequence current is obtained by summing the three-phase currents of each sample.
3. The power frequency components in the zero-sequence current and the frequency components lower than the power frequency are extracted with EMD, and the corresponding IMFs are subtracted from the zero-sequence current signal to obtain the zero-sequence current signal after eliminating the power frequency.
4. According to the mathematical framework of the LSDP method, an LSDP transformation is performed on the zero-sequence current signal of each sample, and the symmetrical image is obtained in polar coordinate space, *r*(*i*), *Θ*(*i*) and *Φ*(*i*).
5. *r*(*i*) is combined with *Θ*(*i*) and *Φ*(*i*) to input the KDE function, calculate the density estimation corresponding to each scatter point and map it to the color map and obtain the LSDP image reflecting the probability distribution density of the scatter point.
6. The training set and the test set are randomly divided, and the average image of the training set LSDP is calculated. The Manhattan distance of the image is obtained by subtracting the average image from a single test image, and this distance is used as a judgment index for fault detection.

## 3. Experimental Conditions and Results Analysis

### 3.1. Experimental Platform and Acquisition Conditions

**Figure 7:**
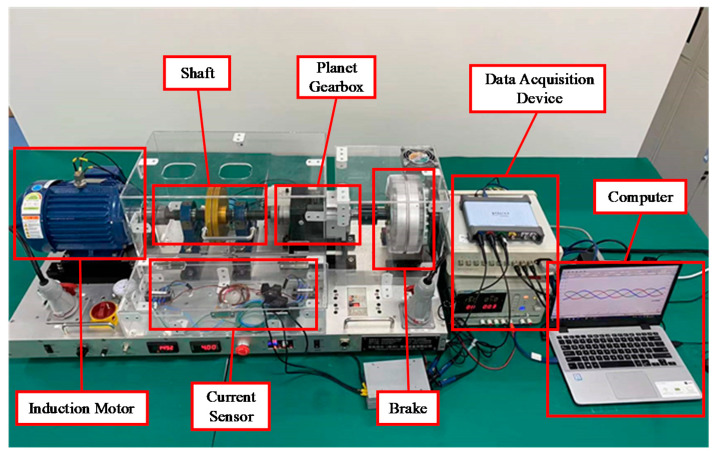
VALENIAN-PT600 motor fault simulation platform.

All of the motor current signal acquisition is carried out on the VALENIAN-PT600 motor fault simulation platform, as shown in Figure 7. The experimental platform consists of two parts: a driving load system and data acquisition system, including the motor, drive shaft, planetary gearbox, frequency converter, magnetic powder brake, current sensor, signal acquisition and conditioning equipment, and computer. The main parameters of the motor are shown in Table 1.

In the experiment, the current data of four typical faulty motors with three different loads are collected, and four typical fault states are simulated:

- Normal.
- Broken bar; four rotor bars in the motor rotor are cut to simulate a broken bar fault while compensating masses are added to correct the resulting unbalanced characteristics.
- Winding short circuit; one stator winding coil is drawn out of the motor junction box and connected to simulate a short circuit.
- Bearing fault; in order to simplify the experimental conditions, the healthy bearings on both sides of the motor are replaced with faulty bearings, in which the fan end is the inner ring faulty bearing, and the drive end is the outer ring faulty bearing.

The signals for all motor fault states are obtained for three different load conditions (0 HP at no load, 1 HP at half load, and 2 HP at full load), so 12 different cases are included in the dataset. The sampling frequency of the acquisition system is set to 200 kHz, and 200 samples are collected for each fault state, each lasting 0.25 s (50,000 sampling points), so there are 2400 samples in total.

### 3.2. Zero-Sequence Current Analysis and EMD De-Power Frequency Results

**Figure 8:**
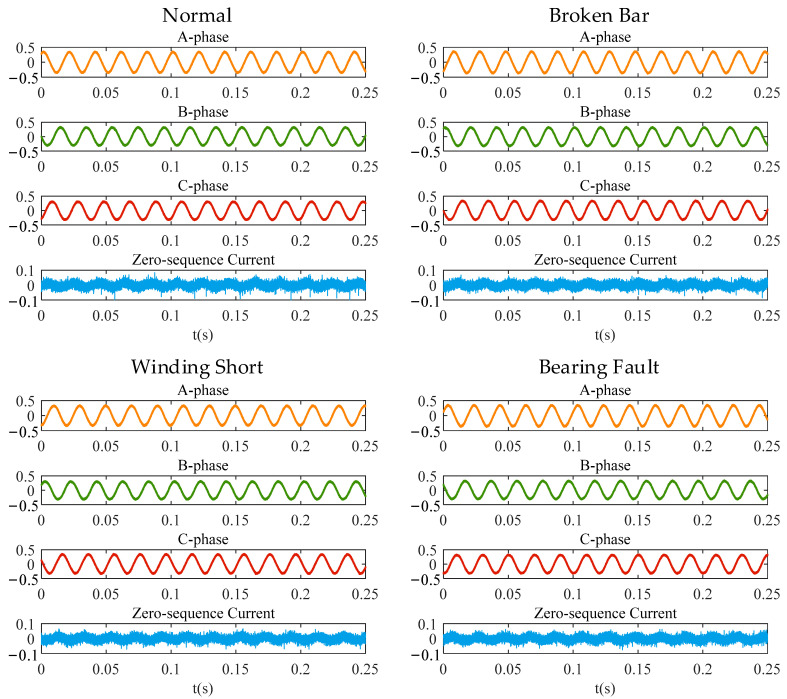
Three-phase current and zero sequence current in each state.

**Figure 9:**
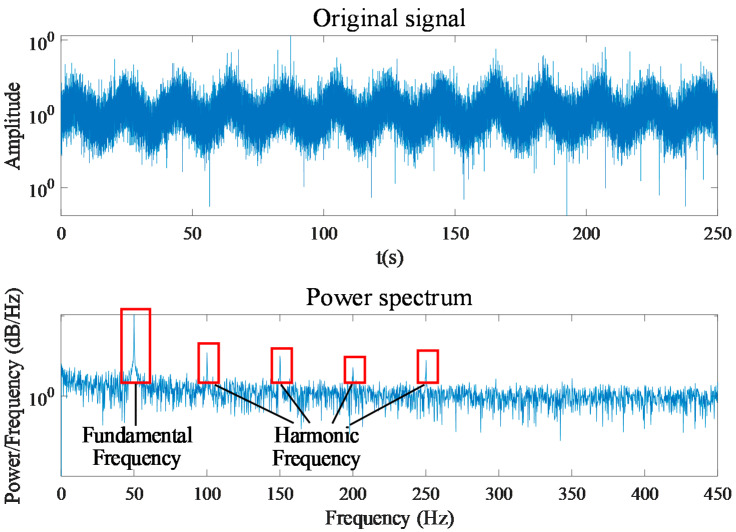
Zero-sequence current and corresponding power spectrum.

**Figure 10:**
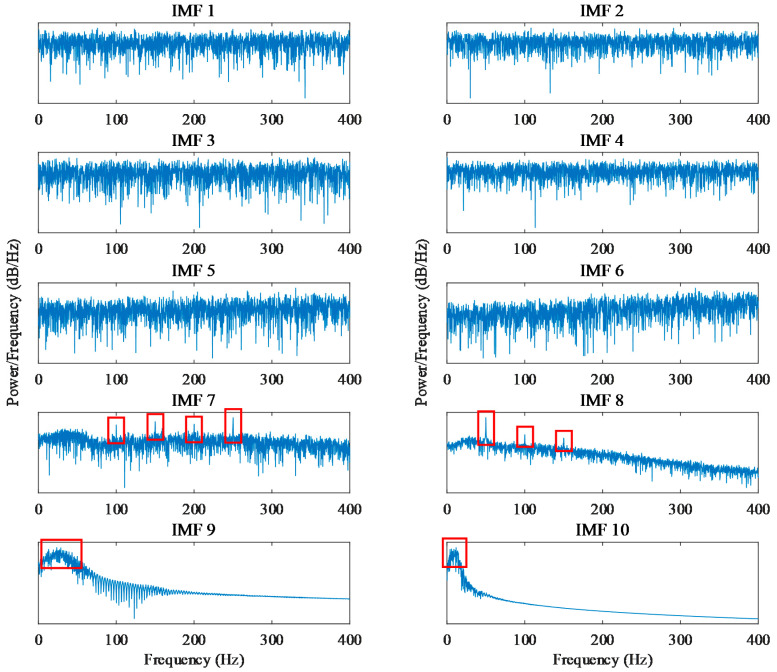
Power spectrum of each IMF for zero-sequence current.

**Figure 11:**
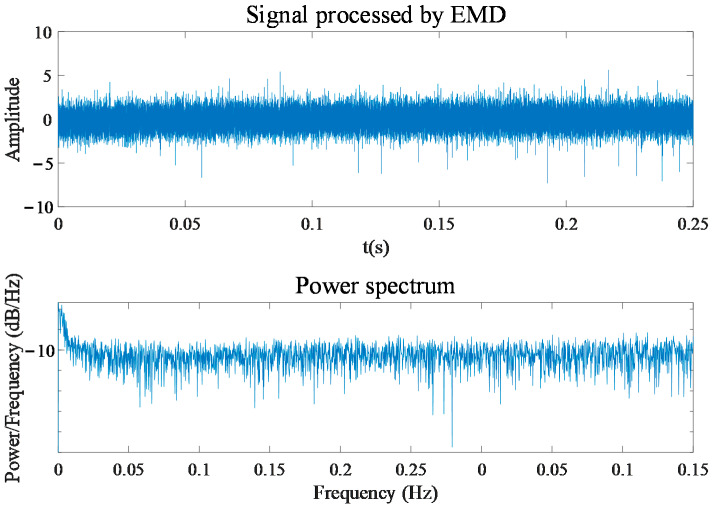
Zero-sequence current and corresponding power spectrum after eliminating power frequency.

According to the implementation procedure proposed in Section 2, the zero-sequence components are obtained by summing the three-phase current signals. It can be seen in Figure 8 that the time-series signal of the zero-sequence current contains the power frequency of 50 Hz and cannot reflect the obvious fault characteristics. The power frequency and its harmonics are more clearly reflected in the zero-sequence current power spectrum shown in Figure 9. By decomposing the zero-sequence current with the EMD method, the IMF components in multiple frequency ranges can be obtained. Taking the zero-sequence current of the motor in the normal state as an example, Figure 10 shows the power spectrum corresponding to each IMF component obtained with decomposition. The frequency components of the first to sixth IMF components are complex, and their energy is concentrated in the high-frequency components, while the energy of the seventh and eighth IMF components is mainly concentrated in 50 Hz and its multiplication frequency. Because the fault characteristic frequency of the motor is generally higher than the power frequency, the influence of the power frequency component and the low-frequency component on the detection result can be removed by subtracting the last four IMF components from the original zero-sequence current signal. Figure 11 shows the zero-sequence current and its power spectrum after eliminating the power frequency. It can be seen that the power frequency component has been removed, but there is still no obvious fault characteristic frequency in the power spectrum.

### 3.3. Comparison of the SDP Method and the LSDP Method

**Figure 12:**
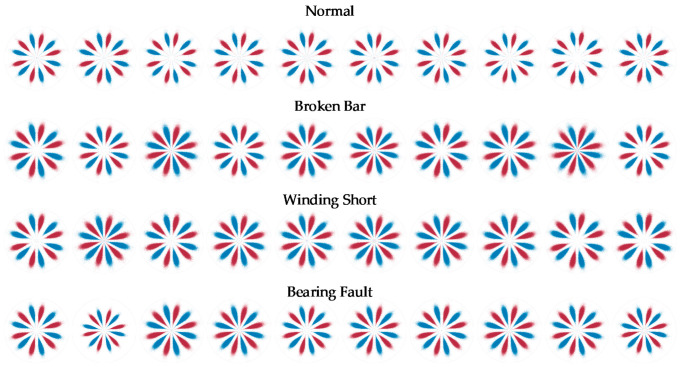
SDP images of samples under full load conditions.

The setting $\theta =60^{{}^{\circ}}$, $g=30^{{}^{\circ}}$, according to Formulas (1)–(3), the SDP coordinates *r*(*i*), *Θ*(*i*) and *Φ*(*i*) can be obtained and drawn in polar coordinates. Figure 12 shows the SDP images of some samples under full load conditions. It can be seen that the scatter distribution of the normal samples is further away from the polar coordinate center than the other three types of faults, but there are several fault samples whose distribution is close to the normal samples, which is infaust to the distinction between normal samples and fault samples. In addition, as mentioned above, due to the interference of wild points, the size of the classic SDP image has obvious fluctuations.

**Figure 13:**
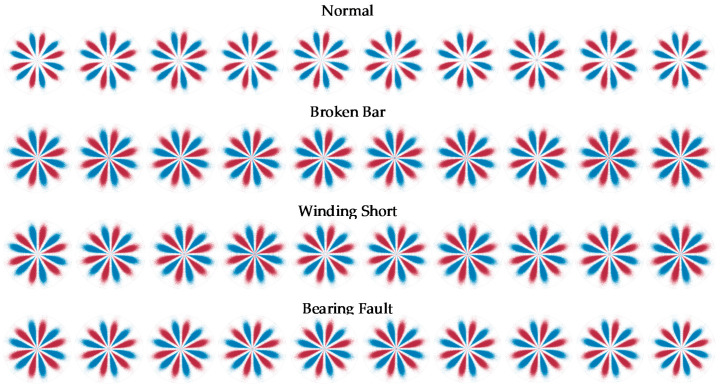
LSDP images of samples under full load conditions.

The setting $\theta =60^{{}^{\circ}}$, $g=30^{{}^{\circ}}$ and *h* = 4000, the LSDP coordinates *r_L_*(*i*), *Θ_L_*(*i*) and *Φ_L_*(*i*) are calculated with Equations (4)–(7) and plotted in polar coordinates. Corresponding to the samples illustrated in Figure 12 and Figure 13 show the LSDP images for the same partial sample set with the full load conditions. Compared with the SDP image, the LSDP image is more stable in size and has better distinguishability for samples that are difficult to define by the SDP, such as the last sample of the broken bar in Figure 12.

**Figure 14:**
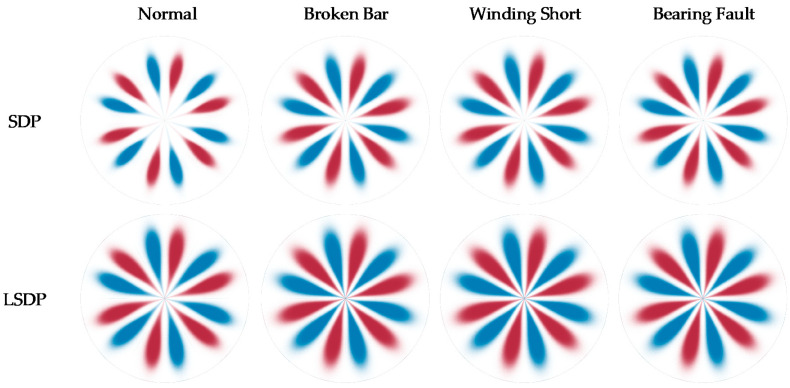
SDP and LSDP average image comparison.

**Figure 15:**
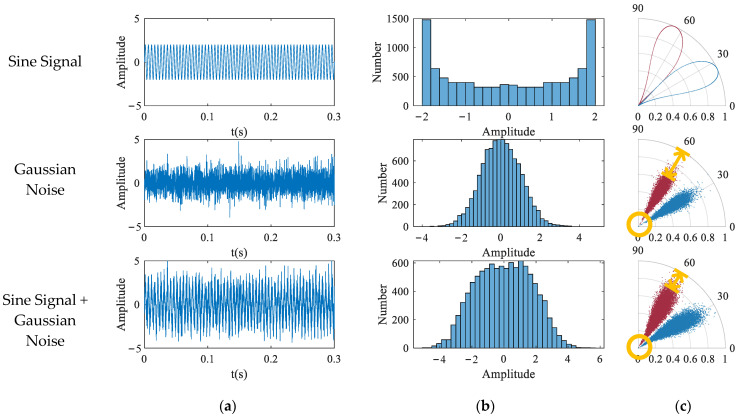
Influence of distribution on SDP image. (**a**) Time domain signal; (**b**) Distribution histogram of sampling points; (**c**) SDP image.

By linearly stacking all sample images at each fault state, Figure 14 shows the comparison between the average images of SDP and LSDP. Although the LSDP sacrifices part of the visually significant difference, the obtained sample images improve the stability. Generally speaking, for the images drawn by the SDP method and the LSDP method, the distribution patterns of the fault types are relatively similar, and the distance from the normal sample to the polar coordinate center is often further than the fault sample, which denotes that the fault characteristics are mainly reflected in the distribution of *r*(*i*). Since both methods have simple linear mappings, it is possible to explain this phenomenon from a distributional point of view easily. This study attempts to mine the fault information hidden in the zero-sequence current through the SDP and its derived methods, which is essential to identifying the fault characteristic frequency submerged in the noise. With the assumption that a Gaussian noise is a measurement noise, the specific frequency sine signal is a fault signal, and its independent distribution and mixture distribution are shown in Figure 15b. The normally distributed noise signal is affected by the fault signal, and the center of gravity of the distribution tends to spread out toward the ends of the range, which is reflected in the SDP polar coordinate image as an increase in the number of points near the origin and boundary. Therefore, when the fault characteristic frequency is present in the zero-sequence current, there are more points near the center of the polar coordinates in the SDP image.

**Figure 16:**
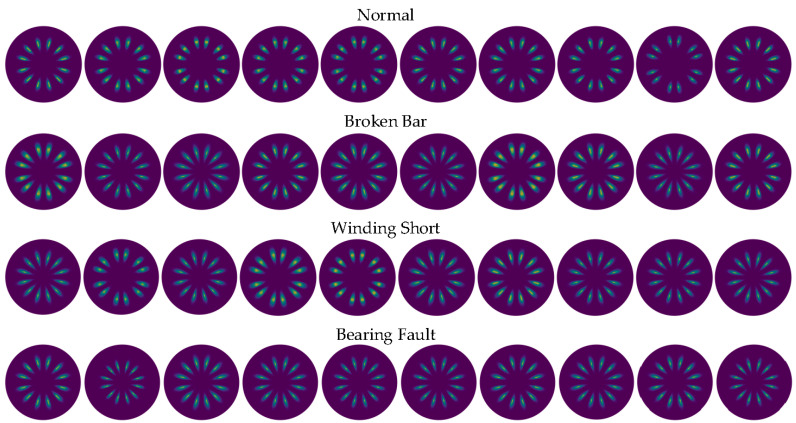
SDP scatter diagram of KDE mapping.

**Figure 17:**
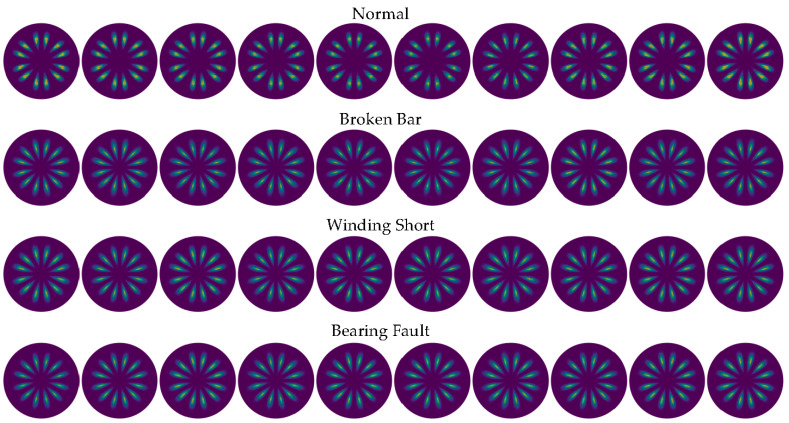
LSDP scatter diagram of KDE mapping.

**Figure 18:**
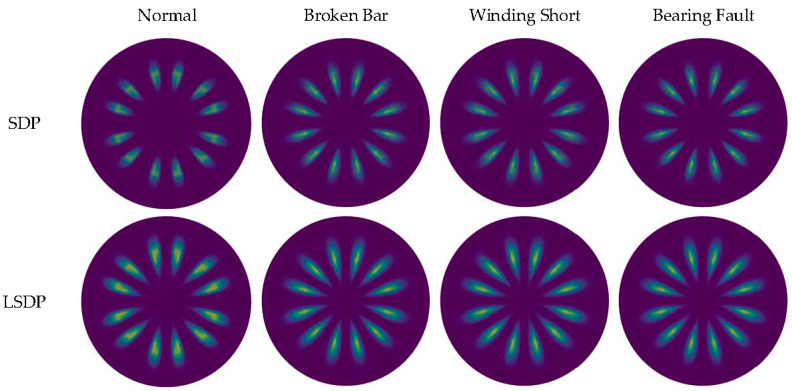
Average image comparison for KDE mapping.

By substituting the polar coordinate data obtained by the SDP and the LSDP into the KDE function given in Equation (10), after obtaining the probability density estimation value corresponding to each sample point, the SDP and LSDP scatter diagrams with colormaps can be redrawn, as shown in Figure 16 and Figure 17. Under full load conditions, there are still obvious fluctuations in the size and distribution pattern of the normal sample and three types of faults in the SDP scatter image mapped using KDE. The scatter density image estimated with the LSDP and the kernel density has more consistent distribution characteristics. The difference in the stability between the two methods is more pronounced in the averaged images, as shown in Figure 18. The average image brightness of the LSDP scatter density is higher with sharper edges.

### 3.4. Fault Detection by Image Distance

The sample images under various working conditions are randomly divided into training/test sets according to the proportion of 75/25, in which the training set is used to calculate the average image. The Manhattan distance from the sample image to the average image is directly obtained from the sum of the absolute values of the RGB differences of the corresponding pixels. For an *m* × *n* image, the Manhattan distance formula is

(12) $$d_{Manhattan}=\sum_{i=1}^{m}\sum_{j=1}^{n}\left|x_{ij}-y_{ij}\right|$$

where *x_ij_*, *y_ij_* are the values of the *i*th row and *j*th column of the two image matrices, respectively.

**Figure 19:**
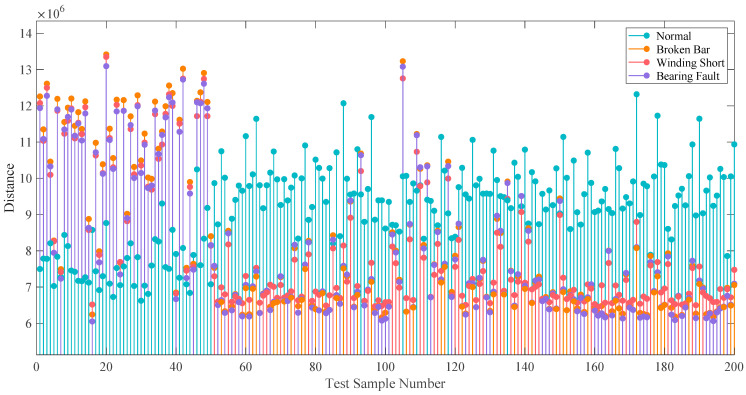
Manhattan distance from sample image to average image.

**Figure 20:**
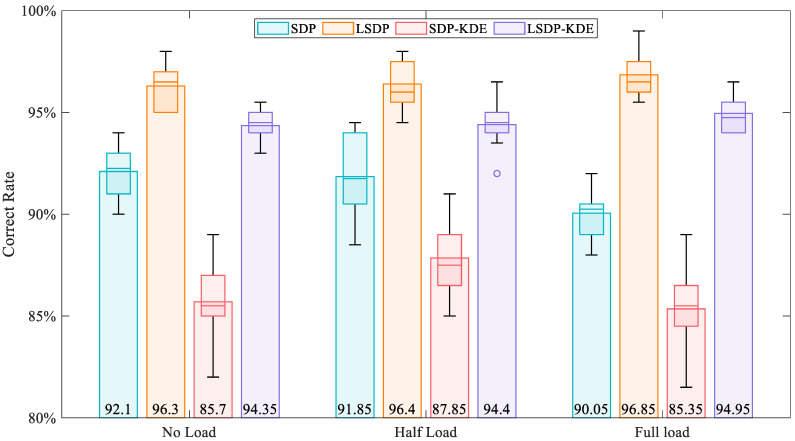
Histogram boxplot for fault detection using Manhattan distance.

For the full load condition of 200 sample data (800 samples in total) in each fault state, four average images are calculated according to the 150 sample images in each fault state, and the Manhattan distance is calculated for the remaining 200 sample images. Figure 19 shows the needle diagram of the Manhattan distance from the 200 sample images of the LSDP-KDE method to the average image under the full load, for which the first 50 samples are normal. Since the distribution and the shape of normal and fault samples in the scatter diagram have obvious reflected differences, a reliable index reference can be obtained through simple image distance calculation. By randomly selecting 75% of the images as the material for calculating the average image and the remaining images as the test samples, fault detection accuracy is investigated. The fault state of the test image is determined according to the minimum distance between the sample image and the four average images. Ten tests are conducted for a single working condition and method. The statistical analysis and the expected value are shown in Figure 20 and Table 2. Comparing the SDP and LSDP methods, the detection accuracy of the LSDP method is more than 4% higher than that of the SDP method under the same conditions. The average accuracy of the LSDP method can reach 96.85% under the full load and 96.3% and 96.4% under no-load and the half load, respectively, indicating that the LSDP has high effectiveness under different working conditions. It is worth noting that although the method combined with KDE makes the visual difference of the images more obvious, the detection effect is reduced. The problem may be caused by the image distance metrics that are used being insensitive to differences in color and shape. Capturing these features using machine learning-based image recognition has the potential to improve the situation. In terms of distribution, the ability of the LSDP method to improve the robustness of fault detection is reflected in both the more compact box lines in Figure 20 and the smaller standard deviations in Table 2. It suggests that the SDP and SDP-KDE methods are more influenced by sample selection, validating the previous analysis of SDP image instability. Overall, the combination with the LSDP method can obtain better fault detection results.

## 4. Conclusions

In response to the shortage of easy-to-understand feature extraction methods for various signal analysis techniques, this paper introduces a visual fault detection method that combines the LSDP and a zero-sequence current. First, the fundamental frequency and low-frequency IMF components in the zero-sequence current are subtracted with EMD to eliminate the influence of the fundamental frequency in the original signal. Then, the time series of zero sequence current is mapped to polar coordinates with the LSDP method, and the point density is estimated using the KDE method and mapped to a color map to obtain the sample image with more apparent brightness and edge features. Finally, the Manhattan distance between the sample image and the average image is used to detect whether there is a fault.

The test results show that the LSDP method has a better adaptive capability for outlier points than the classical SDP method. The correct rate of fault detection with the Manhattan distance can reach up to 99%, and the fault detection rate under different working conditions is no less than 92%.

This method analyzes the fault characteristics by visualizing the zero-sequence current and obtains the distribution shape difference between the normal and fault samples. The use of the KDE method further reinforces this difference while having the potential to apply machine learning for image recognition. Compared with the SDP, the LSDP method has better robustness and stability, and it is more effective in detecting motor faults under different operating conditions. Based on this research, our next work will use SDP images to enrich the representation of motor current information, combine the advantages of machine learning in image recognition to further improve the detection performance, and explore the possibility of classifying faults with these methods. What is more, we believe that this method still has space for advancement in robustness and feature enhancement, so we will also focus on further improvement of the method in future work.

## Figures and Tables

**Table 1:** Induction motor parameters.

Power	Poles	Voltage	Current	Supply Frequency	Speed
1.5 kW	4	220/380 V	6.4/3.7 A	50 Hz	1500 r/min

**Table 2:** Correct rate and its standard deviation for fault detection using Manhattan distance.

Working Condition		SDP	LSDP	SDP-KDE	LSDP-KDE
No load	average	92.10%	96.30%	85.70%	94.35%
	standard deviation	0.0124	0.0116	0.0203	0.0075
Half load	average	91.85%	96.40%	87.85%	94.40%
	standard deviation	0.0200	0.0124	0.0176	0.0117
Full load	average	90.05%	96.85	85.35%	94.95%
	standard deviation	0.0134	0.0106	0.0199	0.0090
